# The importance of living well now and relationships: A qualitative study of the barriers and enablers to engaging frail elders with advance care planning

**DOI:** 10.1177/02692163211013260

**Published:** 2021-05-03

**Authors:** Sarah Combes, Karen Gillett, Christine Norton, Caroline Jane Nicholson

**Affiliations:** 1 Florence Nightingale Faculty of Nursing, Midwifery and Palliative Care, King’s College London, London, UK; 2 St Christopher’s Hospice, London, UK; 3 Faculty of Health and Medical Sciences, Surrey University, Guildford, UK

**Keywords:** Advance care planning, frail elderly, communication, end-of-life care, palliative care, qualitative research

## Abstract

**Background:**

The population of frail elders is growing, and due to their vulnerability to sudden deterioration, advance care planning is particularly important. However, advance care planning is uncommon for multiple reasons, some of which are linked to the perceptions of frail elders and their families.

**Aim:**

To explore the barriers and enablers to advance care planning engagement with frail elders.

**Design:**

Qualitative in-depth interviews with thematic analysis.

**Setting/participants:**

Purposive sample of 10 frail elders and 8 nominated family members using a community-based older persons’ service run by a large urban UK hospice. Frail elders had capacity, were ⩾65 (median 85, range 71–95), scored 6 or 7 (median 6.5) on the Clinical Frailty Scale, and 70% were female.

**Results:**

Key barriers were: *Advance care planning is unclear*, in terms of meaning and the language used; *Lack of relevance*, with frail elders preferring to focus on living well now; and the *Importance of family, relationships and home*, and the influence of relationship on end of life decision-making. Engagement strategies included preparing the frail elder for advance care planning conversations and using a gentle, honest, individualised approach.

**Conclusions:**

Essential enablers for frail elders are understanding what advance care planning is and why it may be relevant to them. For professionals, enablers include recognising the importance of living well now and relational decision-making. To further support advance care planning, recommendations include early engagement and re-conceptualising advance care planning as an ongoing process which encompasses current and future care. Further research is needed in different cultures and care contexts.

**What is already known about the topic?**

- Frailty is increasing worldwide; older people living with frailty are predicted to become one of the largest future users of palliative care.
- Frail elders are at high risk of sudden deterioration and fluctuating mental and physical capacity, meaning advance care planning is particularly important.
- Multiple complex challenges mean advance care planning is relatively uncommon with frail elders; some of these challenges are linked to the perceptions of frail elders and their families.

**What this paper adds?**

- Frail elders and their families can find the concept of advance care planning unclear, with many frail elders believing it is not relevant to them.
- Living well now and family relationships are of far greater importance than advance care planning to frail elders, implying advance care planning should incorporate current as well as future care planning, and recognise the importance of relationships in decision-making.
- Frail elders can be supported to engage with advance care planning by professionals preparing them for conversations, and using an honest, clear, and individualised approach.

**Implications for practice, theory or policy**

- Preparing frail elders for advance care planning conversations and approaching those conversations in a gentle but frank way, provides greater opportunities to increase advance care planning understanding and recognise its relevance.
- Re-conceptualising advance care planning to include current as well as future care would relate more readily to frail elders’ lives.
- Encouraging family involvement, where relevant, and early engagement would recognise the importance of relational decision-making and provide the greatest opportunity for frail elders to engage in advance care planning.

## Background

Frailty affects around 11% of community dwelling adults aged 65 or above.^1^ Its prevalence increases with age^1^ and is expected to increase globally due to population aging.^2^ Characterised by an accumulation of physical, psychological and/or social deficits,^3^ frail elders are more vulnerable to sudden deterioration, reduced recovery potential and increased mortality.^4–7^ Predicted to become one of the largest users of palliative care,^8^ many frail elders receive suboptimal end-of-life care.^9^

Advance care planning enables people to articulate and record their preferences for future care.^10,11^ This supports person-centred care and may reduce inappropriate hospital admissions and invasive, often futile, interventions.^12,13^ Frail elders’ potential for sudden deterioration and fluctuating mental and physical capacity^7,14^ mean advance care planning is particularly relevant. However, advance care planning is relatively uncommon for multiple reasons,^12,15–17^ meaning many frail elders have not discussed their preferences prior to deterioration, when they are less able to engage in decision-making.^14,18,19^

To help frail elders engage with advance care planning, this study aimed to explore the barriers and enablers to advance care planning engagement through better understanding how advance care planning, was viewed by frail elders and their families, who are important influencers of care.^20^

## Methods

### Design

The research questions asked:

- What are the experiences and self-reported attitudes of frail elders and significant others regarding advance care planning?
- What are the barriers and enablers to advance care planning engagement for frail elders?

This was a qualitative study using in-depth semi-structured interviews. Interviews aimed to explore participants’ thoughts and experiences of advance care planning, and their engagement with professionals and others.

### Setting and selection of participants

Participants were purposively sampled from a community-based older persons’ service run by a large urban UK hospice. See Table 1 for inclusion criteria. Participants who met these criteria were then selected to represent those who had and had not formally engaged with advance care planning either as part of the community-based service or through their healthcare professional.

**Table 1.** Inclusion criteria.

Frail elder: Age ⩾65; Rockwood Clinical Frailty Score^a^ (CFS) 6 or 7; Clinically assessed as likely to be in their last years of life; Living in a domestic dwelling; Able to understand English well enough to consent and participate; Has capacity and is assessed as able to cope physically, cognitively and emotionally by their clinician using the Mental Capacity Act or the researcher using the processual model of consent^b^;^21,22^ If bereaved, this should be more than 6 months before recruitment.	Family: Aged 18 or above; Nominated by the frail elder or the referring clinician; Able to understand English well enough to consent and participate; If bereaved, this should be more than 6 months before recruitment.

a The CFS^23^ is scored from 0 to 9. Higher scores representing greater levels of frailty. Older people scoring 6 (moderately frail, require help with all outside activities and keeping house) or 7 (severely frail, completely dependent for personal care) were eligible.

b The processual model of consent^22^ recognises the need to ensure the understanding and decision-making capacity of participants throughout the research in a flexible and individualised way, to enable participants to function at their best capacity.

### Recruitment

Potential participants were initially approached by hospice staff and given a study information sheet. Participants who gave consent to be contacted were approached by the researcher by telephone to arrange an interview.

### Data collection

Interviews occurred between June and September 2019. In all cases participants chose to be interviewed in their home. The topic guide (Supplemental Material 1) was informed by a previous systematic review,^17^ refined with patient and public involvement (PPI) and piloted before use. Probes and supplementary questions were used to clarify meaning and attain more comprehensive responses. The term ‘older people’ was used throughout the study rather than the term ‘frail’ or ‘frailty’. This is because frailty is often understood to have negative connotations, and is not a term that frail elders would recognise within themselves.^24^ Interviews were audio recorded with participants’ consent, transcribed verbatim and anonymised before analysis. Interviews were conducted by a female researcher (S.C.), a nurse with interview training and experience. Participants were aware of the researcher’s background but this was not discussed during the interviews. A reflexive diary was used contemporaneously to prompt insights throughout the study. One insight, following the first three interviews, was that the concept of advance care planning for frail elders was unclear and required considerable explanation. This insight led to the development of vignettes^25^ with PPI (Supplemental Material 2). These vignettes were read by, or to, the following 15 participants as a way to help contextualise the concept of advance care planning. This more creative approach supported conceptual understanding and increased engagement.

### Analysis

Interviews were analysed thematically.^26^ NVivo 12 (QSR International (UK) Ltd) was used to organise data. Transcripts were read and re-read while listening to the recordings, and reading the reflexive diary, to aid data immersion and interpretation.^27^ As this study sought to establish the enablers and barriers to advance care planning, analysis was underpinned by a behavioural change theory.^28^ Therefore, following inductive coding, data were coded deductively by being mapped to a coding frame developed from a previous review^17^ which had used the same behaviour change theory to establish system-wide enablers and barriers for frail elders. The coding frame was expanded to reflect the new themes. Another researcher (C.J.N.) double-coded 10% of transcripts and checked the expanded coding frame to ensure fit. The interpretation, final themes and mapping was discussed and agreed with the research team.

### Patient and public involvement

Frail elders, carers, professionals and voluntary sector representatives constituted an Advisory Group. Communi-cation was face-to-face, by telephone, email and videoconferencing. Contributions included protocol refinement, development of study materials including vignettes and validation of key findings.

### Governance

The study received ethical approval from the North West Greater Manchester Central Research Ethics Committee (reference 19/NW/0148, February 2019), and study site approval (April 2019). The study is reported using the COREQ guidelines^29^ and GRIPP2-SF checklist^30^ for PPI (Supplemental Materials 3 and 4).

## Results

Ten frail elders and eight family members were interviewed (Table 2). Three family participants were related to frail elders who were interviewed in the study. Two daughters of a frail elder (not interviewed) chose to be interviewed together, all other interviews were individual. Interviews lasted a median of 52 min (range 17–84 min).

**Table 2.** Participant characteristics.

Characteristic	Participants (*n* = 18)
**Frail elders interviewed**	*n* = 10
Age	Median 85, range 71–95
Clinical frailty scale (CFS) score	CFS 6 *n* = 5, CFS 7 *n* = 5
Ethnicity	
Asian/Asian British/Indian	*n* = 1
White British	*n* = 8
White other	*n* = 1
Female gender	*n* = 7
Lives alone	*n* = 6
**Family interviewed**	*n* = 8
Relationship to a frail elder	
Spouse	*n* = 4 (*n* = 3 wives, *n* = 1 husband)
Son/daughter	*n* = 4 (*n* = 3 daughters, *n* = 1 son)
Ethnicity	
White British	*n* = 8
Lives with frail elder	*n* = 3 (*n* = 3 wives)

The main barriers and enablers to engaging frail elders with advance care planning were: ‘*Advance care planning is unclear*’, confusion over what advance care planning was and what it included; ‘*Lack of relevance*’, frail elders’ belief that advance care planning was not currently relevant to them; ‘*Importance of family, relationships and home*’, how frail elders discussed family relationships and the significance of home; and ‘*Engagement strategies*’, strategies participants believed could help frail elders engage with advance care planning (Table 3; Theme descriptions are presented in Supplemental Material 5). The views of frail elders and family members were very similar and thus are presented together below. Any differences are highlighted in the analysis. Pseudonyms are used throughout. Relatives’ relationships are also stated.

**Table 3.** Themes and subthemes.

Themes	Subthemes
Advance care planning is unclear	*Understanding what advance care planning means*
	*Clarity of advance care planning language*
Lack of relevance	*Relevance of advance care planning*
	*No need to plan*
Importance of family, relationships and home	*Family will know*
	*Advance care planning is difficult for family*
	*Importance of home*
Engagement strategies	*Right approach*
	*Prepare*

### Advance care planning is unclear

All participants found the concept of advance care planning unclear, and this lack of clarity formed the background to the interviews. This included difficulty *Understanding what advance care planning means*, such as the interviewer making the subject clear to participants, and led to the creation of the vignettes, as previously discussed in Methods. While the vignettes supported conceptual understanding, some confusion still remained.

Most participants said they had not heard of advance care planning. However, during the interviews it became clear that most had engaged in some form of future planning. This included discussing potential equipment that may be required to support them to stay at home, completing a power of attorney, or for twelve participants, documenting the patient’s resuscitation status:

Researcher *‘Have you ever had conversations with anybody about thoughts and feelings towards the end of life or advance care planning?’*

Anne *‘I have mentioned it to family but, that’s as far as it goes.’*

Researcher *‘So nothing with professionals or. . .?’*

Anne *‘No. No.’*

Researcher *‘And is there a reason you chose. . .’*

Anne: *‘I do have a “do not resuscitate” with [hospice].’*

When asked what they thought advance care planning might be, some frail elders believed advance care planning focused only on preparing for death, for example deciding a preferred place of death or funeral arrangements. Others thought advance care planning focused on life now such as their care needs, particularly needs regarding activities of daily living:

*‘Well I’ve heard the name [advance care planning] but I’ve no idea, other than guess work, what it is. [Researcher: What would your guess work be?] Oh, about if you die, where you want to be and that kind of thing?’*

(Ivy)

*‘Well until this happened to me and I had to start having care, you don’t think about it [advance care planning]. . . I never experienced anything like it as such until they started to come for me, all these carers and people. And yeah obviously it’s a good idea in lots of ways because lots of people when they get old and helpless, they do need help.’*

(Elsie)

For many family members though, advance care planning was seen as something broader, encompassing current and future care planning, family, the environment and equipment. For some family members it also encompassed training or information for them, in the form of a tailored booklet for example, so that they could prepare themselves to manage deterioration, or to make ‘*the appropriate decisions as things arise*’ (Carol and Lisa, daughters):

*‘For me it [advance care planning] conjures up, helping a person understand, in a very gentle way, that they need to plan their future, because they obviously have something that’s seriously wrong with them and as we know, it’s not just the person who’s unwell, it’s the family and everyone and. . . . . .you’re actually planning towards, not the very end, but sort of from now, step by step, from now to what is the end, whenever that may be.’*

(Helen, wife)

*‘What happens when he can’t catch his breath? What do I do? That is a big unknown for me and I feel, as his wife and carer, I need to know that. . . I think a lot of carers have that same problem. They don’t know what to do when it gets really, really awful. . . And I find that planning for end of life, or whatever it might be, I find that really, really hard, because I’d like to know what I can do to alleviate the problem.’*

(Lyn, wife)

The lack of clarity regarding advance care planning was compounded by the *Clarity of advance care planning language*. This included unclear documentation and terminology, but also ambiguous professional language which could influence the frail elder’s advance care planning engagement and their trust in professionals. In James’ case, ambiguity led to anger and frustration, and will likely influence any future advance care planning discussions.

*‘I thought he was giving me a choice, and he wasn’t giving me a choice at all, so I don’t really know why he asked the question. He should’ve explained it a different way, you know what I mean? [If] he [had] said “in your present condition, the advice is that we can’t do this [cardiopulmonary resuscitation] because it would do more damage than good.” That, I would’ve understood, but to say “Would you like to be resuscitated?” “Yes please!”’*

(James)

### Lack of relevance

While most family members understood the benefits of advance care planning, many frail elders did not see the *Relevance of* advance care planning to them, and therefore believed there was *No need to plan*. Some appeared ambivalent to advance care planning, and some were not ready to engage, but could see the benefits of advance care planning for other people. For some frail elders, this was because they did not believe they were old enough, or ill enough, to be thinking of future plans. This seemed to be despite, or perhaps because, they had experienced and to some degree recovered from, deteriorations. Other participants appeared to recognise the uncertainty of frailty, but believed advance care planning was futile due to potentially rapid health changes.

*‘I haven’t really felt the need for it as yet. And I’ve only just reached 80. I was feeling unwell a couple of weeks ago, to the point where I was thinking I was on my way out, but it turned out to be a couple of infections that I had that treated with antibiotics cleared up in very short time.’*

(Ernest)

*‘I think planning for the future, there are so many different possible scenarios that, how can you? I think you just have to be adaptive as you go along. . . .they [frail elders] do look day by day, in fact they look hour by hour. You know particularly as the mind becomes slightly less reliable, they’re certainly not going to be looking forward and trying to look at this scenario and that scenario.’*

(Simon, son)

Many frail elders spontaneously expressed future care preferences throughout the interviews. However, they also appeared ambivalent to engaging with advance care planning. For many frail elders this seemed to be due to previous experiences, such having a health, social care or legal background, experiencing poor care, or because they believed someone else would make any final decisions (see *Family will know*).

*‘Well, care planning, a lot of people want to dot the “I’s,” cross the “T’s” and want to make sure that you’re going to go into hospital or you’re going to go to [hospice], and you’re going to be looked after. And sometimes it happens and sometimes, it doesn’t happen. I mean you make all the plans in the world and you can do what my son did, get out the car and drop dead. . . There’s no guarantee that it is going to happen, and this is where my carer comes in. I’m afraid she’s right. . . what will be will be. What’s the song say? Que sera, sera [what will be, will be].’*

(Dorothy)

Many other frail elders appeared ambivalent to advance care planning as they saw death and dying as part of life and did not want to ‘waste time’ planning for an uncertain future. Despite this, all seemed comfortable talking about advance care planning, with frail elders often using humour if the conversation became emotional. This appeared to the researcher to be a coping strategy to lighten the mood and help ease any tension felt while talking about their own mortality.

*‘Somebody said to me the other day about thoughts of the future and I thought, well, only, erm, this makes me a bit weepy; not because I’m sad but it’s just talking about it. Erm, the future - the only future is erm, getting ill or collapsing and dying [laughter]. I mean er, that sounds charming, doesn’t it? But that really is the future.’*

(Ivy)

*‘People have to die. Nobody probably, I never wanted my mum, especially my poor little old mum to die, but they have to eventually, nothing you can do about it, that’s life isn’t it. . . .hopefully I’ll still be here, in my own home, until it happens, and what happens after that won’t interest me because I don’t think there’s no shops up there [laughs].’*

(Elsie)

Overall, participants were far more interested in discussing living well now and maintaining their quality of life than in talking about advance care planning. Living well now included multiple topics such as being listened to, having the right equipment and environment, and being supported by family, friends and professionals. Two elements of living well now that were discussed by all participants, were maintaining or improving mobility and independence, and finding tasks or hobbies that helped them feel valued and useful.

*‘I dread the day that might come, when I will not be able to get out of bed. That’s why I’m doing these exercises quite, earnestly, but that [being confined to bed] really would be the last straw for me.’*

(Grace)

*‘If I can even get up off the chair and walk around with a Zimmer frame, I’d love to make a cup of tea for my wife, that would be it.’*

(Bill)

*‘I’ve got to find whatever I can do, just to feel as though I’m helping out.’*

(Ernest)

### Importance of family, relationships and home

Relationships were important to frail elders and impacted decision-making. While family relationships seemed of upmost relevance to frail elders, relationships with professionals were also important, with good relationships seeming to influence the frail elder’s view of services and end-of-life care preferences. Many frail elders voiced advance care planning preferences during the interviews; however, most then stated they were leaving decisions to their family or professionals. When asked why, frail elders said their *Family will know* what they wanted, would have to live with any decisions, or that they trusted family or professionals to make best interest decisions should the need arise.

*‘They’ve all got their heads screwed on, and they know what’s best, and they’d say, you know, he’s better off this way, better off that way, and I trust them entirely. . .’*

(James)

*‘They don’t get to be professional at their jobs without learning by all their patients’ experiences. And therefore I’m. . . happy to go along with their professionalism and their experience and their ideas.’*

(Ernest)

However, in most cases, frail elders had not discussed their preferences with family. This appeared to be because frail elders believed *Advance care planning is difficult for family*. Reasons included the taboo around death, and not wanting to upset or be an additional burden to, their family. Concern regarding being a burden is demonstrated in the quote below where Ivy describes not telling family her advance care planning preferences for fear of burdening what Ivy sees as her stepdaughter’s already busy life.

Researcher *‘I’m kind of intrigued that you write it [advance care planning decisions] down on bits of paper instead of just telling [stepdaughter].’*

Ivy: *‘Ah, well I only see her once a week and she pops in on her way home from work. She’s got a busy job. She’s got animals to look after and so on, and I - probably I have spoken vaguely about it but. . . I suppose I haven’t talked to her much like I am to you now. . . I sometimes write on a bit of paper, oh next time she comes, I’ll tell her this or I’ll ask her that but I don’t, I do it as least as I can mainly, I think, because I don’t really want to be a burden to her.’*

Concern over advance care planning being difficult led to many frail elders worrying about leaving their family and wanting to support them to prepare for the frail elder’s death. For many frail elders it appeared that advance care planning was more about concerns regarding family than their own mortality.

***‘*** *It’s like, something where you know you’re gonna be alright, in a certain way. But you know some people [family] might struggle. And you don’t want it to spoil their life, thinking about something, you know, that they could have done or not done.’*

(Anne)

*‘Up until a certain point, I would be thinking about him [son] . . . and how he’s coping with it. [Researcher: So looking after him is important.] Mmm, and the grandchildren because they are young. . . .just as long as I know that there’s somebody there to help him as well, afterwards.’*

(Sally)

Family participants agreed that advance care planning was difficult. This seemed to be because they did not want to admit their family member was nearing the end of life. Whether advance care planning happened or not, some family participants felt they knew what the frail elder’s preferences were, whereas others felt unsure or worried that they may not be ‘*making the right decisions*’ (Carol, daughter).

*‘. . .my children don’t want to talk about it, but they will have to talk about it because I don’t want them to go, you know, what we’ve been going through. So at least they know, but you’d never want to talk about things like, well I didn’t, I’d always say “No, I don’t want to talk about it now. You’re going to live forever. [laughs].”’*

(Lisa, daughter)

Many frail elders talked about the *Importance of home* and their desire to remain at home rather than being admitted to hospital or long-term care. Reasons for this included previous poor care experiences and worrying over long-term care costs. But mainly frail elders wanted to remain at home because they would be surrounded by their family, possessions, and everything they knew.

*‘She would have wanted, I know, to be at home, with the things, the photographs, when she was more cognisant she would always be looking at her photographs or coming in here and looking at the diplomas and things, you know, this is where she’d want to be.’*

(Donald, husband)

*‘If I had to have medication or injections, I would rather have it at home. I would rather stay at home as long as possible, and pass away peacefully at home with my wife around me or my family around me.’*

(Bill)

### Engagement strategies

Towards the end of the interviews, participants were asked how we could help engage frail elders with advance care planning. Participants suggestions formed two main themes; using the *Right approach* and helping frail elders *Prepare* for advance care planning conversations. The *Right approach* included advance care planning being led by a professional they knew and trusted, who used gentle, clear language, ensured understanding, and listened to them. It also included advance care planning being treated as normal, everyday conversations, and some frail elders saying they would prefer advance care planning to be discussed in a light-hearted rather than serious way.

*‘It’s been always done very gently, and very normally, it’s not a hush hush, you know, it’s been normal and that’s the criteria I think, to be normal but gentle so that what’s happening to you is part of your life, . . .you can’t go in with hob nail boots, you have to have nice little fluffy slippers [laughs]. . . Well, you know, it is gentleness isn’t it and it’s understanding and listening, literally listening.’*

(Helen, wife)

When asked about taking part in the interview

*‘It’s been quite light-hearted, it’s not miserable. And that’s the way it [advance care planning] should be really.’*

(Edith)

The *Right approach* also comprised advance care planning conversations being honest and frank, such as being truthful about the frail elder’s likely trajectory or potential care or treatment choices, and tailored to each individual, as some frail elders were recognised as requiring more time or support to engage.

‘*I’m fortunate because I do have a medical background if you like, but a lot of people don’t and they don’t know, you know. They don’t know a broken finger from a broken neck but I think that they should. If they think. . . when you’re chatting to them, if you get the inkling that they think, ‘Oh, if I fall down, it’ll be alright. Somebody will fix me up,’ then at that age, I think they should be told, “Well, if you fall down and break your leg. . . it’s possible that you will never walk again.”’*

(Dorothy)

*‘It’s how you actually approach it and go about it is a main point, I think. Some people you’ve got to approach sensitively and others, like me. . . normal conversation, you know.’*

(Sally)

Participants were asked if they thought receiving information would help frail elders *Prepare* for advance care planning. This received various responses. While films or board-games were suggested by two individuals, most supported a written leaflet, but felt its usefulness would depend on whether the individual enjoyed reading. Only two frail elders supported online documentation. In all cases, participants felt written information should act as an adjunct to face-to-face discussions.

*‘I think that would be very, very helpful erm, not just for the patient but also for the family who in the long run have to sort it out.’*

(Sandra, daughter)

*‘I. . . computers are just. . . not within my reasoning at all.’*

(Edith)

*‘I think it’s got to be something that is done personally, by personal contact, perhaps followed up with literature or a booklet, but I don’t believe that a booklet in the first instance would help the majority of people.’*

(Sheila, wife)

The time to *Prepare* for the interviews, between booking and the interview itself, appeared to be appreciated. Six participants stated that while they had not previously thought about advance care planning, they were now interested in engaging. The time to *Prepare* may have been enhanced by the interviewer using a gentle, honest approach alongside vignettes to help people engage more easily, similar to the *Right approach* suggested above.

*‘I hadn’t already thought about the future. But since you’ve come and since I’ve had notification of you coming, I’ve thought about it. I think the fact is that if it does deteriorate, I would still like to be at home with my family. . .*

*[Discussing advance care planning engagement] “I would much prefer somebody like you rather than to read a leaflet or booklet because you’re inclined to skip through the wording and not really take the information in deeply. I find your interview is far better because you’re talking openly and not thinking too much about it until the question is actually put to you.”’*

(Bill)

## Discussion

### Main findings

For most participants advance care planning was unclear, both in terms of meaning and the language used. Frail elders often did not see the relevance of advance care planning for themselves, either because they did not believe they were ill enough to engage or because they preferred to focus on living well now. Relationships were integral and impacted decision-making. Participants believed the best way to engage frail elders with advance care planning was by using the right approach and preparing individuals for advance care planning conversations.

### What this study adds

For frail elders in this study, advance care planning was most aligned with putting affairs in order, what Pollock et al.^15^ would refer to as *personal co-ordination of care*, and current care provision. For family participants, advance care planning was much broader, including current and future care plans, family and the environment. While all frail elders were open to advance care planning conversations, they needed to understand advance care planning’s relevance for them, particularly as most were more interested in living well now, a coping strategy suggested as essential for those nearing the end of life.^31–33^ These findings imply that for advance care planning to be relevant to frail elders it should encompass not just future planning, which can be difficult to imagine when living with uncertainty,^34^ but also planning for now and how things may change. This tension between advance care planning the, often medicalised, documentation and advance care planning the process has been highlighted with the recent covid-19 pandemic.^35^ Our findings support the concept of advance care planning as a process that promotes meaningful relationships and strengthens support for frail elders as they near the end of life.^15,36^ Further it supports the conceptualisation of advance care planning as part of usual care^13,37,38^ with a more flexible, preference-based approach.^39,40^

Relationality and relational decision-making were important for frail elders in our study. While most had advance care planning preferences, they stated they were leaving any decision-making to family or professionals. These findings support literature which suggests that rather than personal autonomy, many older people prioritise relational autonomy,^20,41,42^ which recognises that identity and self-determination are formed through relationships and social situations.^43^ However, frail elders rarely discussed their advance care planning preferences, and while some family participants felt they knew the person’s wishes, others worried about making the ‘right’ decisions, supporting literature that suggests patient and surrogate decision-maker views do not always correspond.^44^ Thus, while Killackey et al.’s^45^ model of relational autonomy recognises personal vulnerability and fluctuating trajectory, it is important for frail elders that this model be expanded to encourage family member engagement.

### Implications for practice

Frail elders had difficulty understanding what advance care planning was and its relevance to them, despite being prepared for advance care planning discussions during the recruitment process. This has implications for achieving shared decision-making and person-centred end-of-life care. Participants’ focus on living well now suggests professionals should begin discussions with what is important to the individual now, and recognise that advance care planning for frail elders encompasses more than medicalised decision-making. This approach supports the concept of advance care planning as a process, which supports the development of trust and rapport between individuals and professionals, cited as the ‘cornerstone’ of advance care planning conversations.^46^ Further, it promotes early engagement, a concept that provides the greatest opportunity for frail elders to engage with advance care planning physically and cognitively.^14,17^

Conversations will need to contextualise advance care planning for individuals who believe they are not old enough or ill enough to engage. This may be very hard to hear and challenge an individual’s coping strategies.^24,47^ Thus, it is recommended that professionals prepare frail elders for advance care planning conversations and use the ‘right approach’. Preparing frail elders may include setting a time and date for initial advance care planning conversations, and providing brief, understandable documentation that can be read and discussed.^17,48^ The approach should be clear, understandable, honest and frank, individualised and should progress at the frail elder’s pace. This may include discussing the individual’s likely trajectory or past health experiences, and using vignettes as a gentler approach to conversations.^25,49^

Given the importance of relational autonomy, professionals should also support family involvement, such as encouraging discussions with family regarding preferences, and suggesting family are present during advance care planning conversations where relevant. This would likely support engagement and reduce the potential for family burden should the individual loose capacity in the future. Exploring appropriate approaches, professional training and documentation for frail elders is a target for future research.

### Ethical considerations

From the ethical perspective, several strategies were implemented to support frail elders to engage in the interviews. Frail elders were asked whether they would prefer to be interviewed alone or with a nominated person, and if they wished the researcher to discuss the study with a family member before giving consent. No participant took up either offer, although on the day of the interview, three husbands suggested their wives remain in the room while they were interviewed. This decision appeared to be more about not wanting their wives to move from where they were comfortable rather than because the interviewee wanted their wife to contribute to the interview. Further, a distress protocol was developed for use if participants became upset. However, while three participants said talking about advance care planning made them sad, none wanted to stop the interview, instead each took a couple of minutes to compose themselves before asking to continue.

### Strengths/limitations

The study collected rich data from a vulnerable group who are little researched due to ethical concerns and methodological challenges.^50^ It was strengthened by PPI which helped support participant engagement and acceptability.^29,51^ Interviews were conducted by one researcher. This enabled a consistent approach and the potential for interpretation bias was mitigated by using a reflexive diary, research team discussions and double-coding. One limitation of the study was the decision to interview only moderately or severely frail elders. Engaging frail elders with advance care planning early is encouraged by the authors. However, this study is part of a larger programme of work focused on supporting advance care planning conversations in practice with those who have the greatest clinical need. Further research is needed regarding barriers and enablers to advance care planning with older people who are pre-frail or mildly frail. Findings from this study have been used to underpin new practice guidelines which are in use at the study site (Supplemental Material 6), and are being used to inform the development of an intervention underpinned by behavioural change theory.^28^ Further research is needed in different cultures and care contexts.

## Conclusions

This paper describes the barriers and enablers to engaging frail elders with advance care planning. Based on these findings, essential enablers for frail elders are understanding what advance care planning is and why it may be relevant to them. For professionals, essential enablers are recognising the emphasis many frail elders place on living well now and relational autonomy rather than autonomous future planning. The paper also presents strategies for engagement suggested by participants. These included preparing the frail elder for advance care planning conversations and using a gentle, honest, individualised approach. To support this, early engagement and the conceptualisation of advance care planning as an ongoing process that includes current and future care is recommended.

## Supplemental Material

sj-pdf-1-pmj-10.1177_02692163211013260 – Supplemental material for The importance of living well now and relationships: A qualitative study of the barriers and enablers to engaging frail elders with advance care planning Click here for additional data file. Supplemental material, sj-pdf-1-pmj-10.1177_02692163211013260 for The importance of living well now and relationships: A qualitative study of the barriers and enablers to engaging frail elders with advance care planning by Sarah Combes, Karen Gillett, Christine Norton and Caroline Jane Nicholson in Palliative Medicine

sj-pdf-2-pmj-10.1177_02692163211013260 – Supplemental material for The importance of living well now and relationships: A qualitative study of the barriers and enablers to engaging frail elders with advance care planning Click here for additional data file. Supplemental material, sj-pdf-2-pmj-10.1177_02692163211013260 for The importance of living well now and relationships: A qualitative study of the barriers and enablers to engaging frail elders with advance care planning by Sarah Combes, Karen Gillett, Christine Norton and Caroline Jane Nicholson in Palliative Medicine

sj-pdf-3-pmj-10.1177_02692163211013260 – Supplemental material for The importance of living well now and relationships: A qualitative study of the barriers and enablers to engaging frail elders with advance care planning Click here for additional data file. Supplemental material, sj-pdf-3-pmj-10.1177_02692163211013260 for The importance of living well now and relationships: A qualitative study of the barriers and enablers to engaging frail elders with advance care planning by Sarah Combes, Karen Gillett, Christine Norton and Caroline Jane Nicholson in Palliative Medicine

sj-pdf-4-pmj-10.1177_02692163211013260 – Supplemental material for The importance of living well now and relationships: A qualitative study of the barriers and enablers to engaging frail elders with advance care planning Click here for additional data file. Supplemental material, sj-pdf-4-pmj-10.1177_02692163211013260 for The importance of living well now and relationships: A qualitative study of the barriers and enablers to engaging frail elders with advance care planning by Sarah Combes, Karen Gillett, Christine Norton and Caroline Jane Nicholson in Palliative Medicine

sj-pdf-5-pmj-10.1177_02692163211013260 – Supplemental material for The importance of living well now and relationships: A qualitative study of the barriers and enablers to engaging frail elders with advance care planning Click here for additional data file. Supplemental material, sj-pdf-5-pmj-10.1177_02692163211013260 for The importance of living well now and relationships: A qualitative study of the barriers and enablers to engaging frail elders with advance care planning by Sarah Combes, Karen Gillett, Christine Norton and Caroline Jane Nicholson in Palliative Medicine

sj-pdf-6-pmj-10.1177_02692163211013260 – Supplemental material for The importance of living well now and relationships: A qualitative study of the barriers and enablers to engaging frail elders with advance care planning Click here for additional data file. Supplemental material, sj-pdf-6-pmj-10.1177_02692163211013260 for The importance of living well now and relationships: A qualitative study of the barriers and enablers to engaging frail elders with advance care planning by Sarah Combes, Karen Gillett, Christine Norton and Caroline Jane Nicholson in Palliative Medicine
