# Supplementary material for: The importance of living well now and relationships: A qualitative study of the barriers and enablers to engaging frail elders with advance care planning
Source: Palliat Med. 2021 May 3;35(6):1137–47. doi: 10.1177/02692163211013260 (PMC8189003; doi:10.1177/02692163211013260)
Supplement: sj-pdf-1-pmj-10.1177_02692163211013260 – Supplemental material for The importance of living well now and relationships: A qualitative study of the barriers and enablers to engaging frail elders with advance care planning [file sj-pdf-1-pmj-10.1177_02692163211013260.pdf]

Supplementary material 1: Topic guide

Family members were asked the same questions but framed from their experiences with frail elders. The term ‘older people’ was used throughout as frail elders do not recognise the term frailty as relevant to themselves:

Understanding of advance care planning

- Have you ever heard of advance care planning?
- What does advance care planning for older people mean to you? (explain/discuss what advance care planning means where required)

Experiences and views

- Explore advance care planning views more deeply using vignettes
- What are other important things to think about or plan for? Or, If someone close to you were in the last year of life, what sort of things do you think would be useful for them to think about or plan for?
- What are the things that you value most when you think about living a good quality life?
- Have you ever had an advance care planning conversation? With whom? Motivation? Feelings?
- When you think about advance care planning, do you think about plans for yourself, or are you thinking about planning for family or friends too?

Suggested strategies to engage older people

- Would it be helpful to receive some form of documentation that helped you start thinking through your advance care planning goals and values before any formal advance care planning conversations with professionals?
- Would you find it helpful if you held onto your advance care planning, so you could maintain it and share it with others?

Ending the interview

- Is there anything else you thought we would discuss today?
- Is there anything else you would like to say/ask, any final thoughts?
