# Supplementary material for: The importance of living well now and relationships: A qualitative study of the barriers and enablers to engaging frail elders with advance care planning
Source: Palliat Med. 2021 May 3;35(6):1137–47. doi: 10.1177/02692163211013260 (PMC8189003; doi:10.1177/02692163211013260)
Supplement: sj-pdf-2-pmj-10.1177_02692163211013260 – Supplemental material for The importance of living well now and relationships: A qualitative study of the barriers and enablers to engaging frail elders with advance care planning [file sj-pdf-2-pmj-10.1177_02692163211013260.pdf]

Supplementary material 2: Vignettes

Participants read, or had read to them depending on preference, two of the three vignettes. The choice of vignettes was based on which the researcher felt would be most likely to elicit a rich discussion with each individual at the time.

Vignette 1: Florence

Florence wanted to remain at home for her care until the end of her life, being supported by her family and the professionals caring for her. But, if her health worsened significantly, she wanted to move to a nursing home. She didn't want to receive cardiopulmonary resuscitation. If she ever needed medical treatment, such as for an infection, she only wanted treatments that could be given in her own home even if that meant it would shorten her life. She had discussed her wishes with her son, who was her main carer, and she had written an advance care plan to let people know her wishes. This was held by her and her GP. Day to day the most important thing for Florence was to support her family and help them cope with life when she had gone, this included writing a will.

Vignette 2: Fred

Fred had been in hospital several times over the last few months. While he would rather not go back again, both Fred and his wife felt it was important for him to be given every opportunity to live for as long as possible. He wanted to receive full, active treatment as needed, including going into intensive care and having a feeding tube if he could not take food by mouth etc. He also wanted to receive cardiopulmonary resuscitation, although he understood this would be a decision made by the clinical team at the time, and may not restore him to good health. For Fred, while he would rather have both quality and quantity of life, quantity was the most important.

Vignette 3: Marjorie

Marjorie didn't want to specify an advance care plan around her future care wishes. She believed what would be would be, trusting her family to make the right decisions if she was ever in a position not to be able to make her own decisions in the future. For Marjorie the important thing was living each day to the full and not wasting the time she had. She focused on "day to day" planning for doing the things she wanted to do, such as deadheading the roses, having a meal out with his family, and carrying out the exercises her care team had given her to help her keep on her feet. She felt this "day to day" planning, which allowed for how she was feeling each day, brought some order to her life.
