# Supplementary material for: The importance of living well now and relationships: A qualitative study of the barriers and enablers to engaging frail elders with advance care planning
Source: Palliat Med. 2021 May 3;35(6):1137–47. doi: 10.1177/02692163211013260 (PMC8189003; doi:10.1177/02692163211013260)
Supplement: sj-pdf-4-pmj-10.1177_02692163211013260 – Supplemental material for The importance of living well now and relationships: A qualitative study of the barriers and enablers to engaging frail elders with advance care planning [file sj-pdf-4-pmj-10.1177_02692163211013260.pdf]

Supplementary material 4: GRIPP2 short form

Section and topic	Item	Reported on page No
1: Aim	Report the aim of PPI in the study	5, 16
2: Methods	Provide a clear description of the methods used for PPI in the study	5
3: Study results	Outcomes—Report the results of PPI in the study, including both positive and negative outcomes	5, 16
4: Discussion and conclusions	Outcomes—Comment on the extent to which PPI influenced the study overall. Describe positive and negative effects	5, 16
5: Reflections/critical perspective	Comment critically on the study, reflecting on the things that went well and those that did not, so others can learn from this experience	16

PPI=patient and public involvement

Staniszewska S, Brett J, Simera I, et al. GRIPP2 reporting checklists: tools to improve reporting of patient and public involvement in research. Research involvement and engagement 2017; 3: 13.
