# Supplementary material for: The importance of living well now and relationships: A qualitative study of the barriers and enablers to engaging frail elders with advance care planning
Source: Palliat Med. 2021 May 3;35(6):1137–47. doi: 10.1177/02692163211013260 (PMC8189003; doi:10.1177/02692163211013260)
Supplement: sj-pdf-5-pmj-10.1177_02692163211013260 – Supplemental material for The importance of living well now and relationships: A qualitative study of the barriers and enablers to engaging frail elders with advance care planning [file sj-pdf-5-pmj-10.1177_02692163211013260.pdf]

Supplementary material 5: Theme descriptions

Themes and subthemes	Description
**Advance care planning is unclear:**	Most participants were confused regarding advance care planning. Many had not heard of advance care planning, suggestions for what it may include were often confused with other planning, although often some form of advance care planning had taken place. This was compounded by lack of clarity around advance care planning language.
Understanding what advance care planning means	Advance care planning was not well understood and often confused with other forms of planning, although some had made future plans.
Advance care planning is getting things in order	Advance care planning conceptualised as getting things in order for death and/or dying e.g. funerals, preferred place of death.
Advance care planning is current care provision	Advance care planning conceptualised as current care provision, particularly social care.
Advance care planning is more than planning for death and dying	Advance care planning conceptualised as current as well as future care planning. Includes family, environment, equipment, and training/information on managing care as frail elder deteriorates.
Making plans	Formal and informal future plans the frail elder had made.
Clarity of advance care planning language	Language used to express and explain advance care planning is unclear.
Terminology/Documentation	Multiple documents and concepts that were unclear e.g. living wills, lasting power of attorney, do not attempt resuscitation orders and advance care planning.
Unclear language	Where participants were confused by professionals' choice of language.
**Lack of relevance**:	Where participants could not see relevance of advance care planning to them personally/the frail elder. It was a waste of time as trajectories are uncertain or that the concept of planning for their death/dying was not as important to them as, for example, living well now.
No need to plan	Reasons given for why advance care planning was not of relevance to the participants/the frail elder.
I don't need to yet	The person feels they are not old enough/ill enough for advance care planning to be required.
It's difficult to plan when you live with uncertainty	Frailty's uncertain trajectory made making future plans difficult.
Relevance of advance care planning	Participants were more interested in living well day to day and maintaining their current quality of life rather than future planning.
I'm more interested in living well now	Where frail elders focussed on quality of life day to day rather than future planning.
Death as part of life	Death and dying seen as part of life, death acceptance, humour use, taboo as well as appropriate sadness.

Themes and subthemes	Description
**Importance of family, relationships and home**	Where frail elders showed more concern for the relationship with and protecting their family than their own end of life journey.
Advance care planning is difficult for family	Where frail elders found difficulty speaking with their family about advance care planning. Family members also found these conversations difficult and avoided them.
Family will know	Most frail elders expressed preferences for advance care planning but believed their family, or professionals, would make all final decisions as their family knew what they would prefer, although they may never have told them. Family often did not know the person's preferences.
Importance of home	Priority frail elders put on remaining at home with those they loved and their own things around them.
**Engagement strategies**	Behaviours and strategies participants felt could encourage frail elders to engage with advance care planning.
Right approach	The approach participants believed would help frail elders engage with advance care planning.
Conversational approach	Advance care planning conversations should be treated like normal, everyday conversations, delivered with a light-hearted rather than serious approach.
Be gentle and listen	This included using a gentle approach, such as introducing advance care planning through vignettes or reminiscence work, and listening to what the frail elder had to say.
Tailor the approach to the individual	This included professionals assessing how ready a frail elder was to talk about advance care planning and progressing conversations at the frail elder's pace.
Be honest	This included professionals being honest about the frail elder's likely trajectory, as well as any potential care or medical treatment choices. Also included how lack of honesty could impact trust of professionals, services and advance care planning.
Prepare	The relevance of preparation to informed advance care planning conversations.
Time	This includes how frail elders found being given time to plan for advance care planning, gave them time to think about their preferences, and sometimes discuss it with others, and helped them engage with conversations.
Resources	This includes suggestions of leaflets, booklets, films and board-games. Online information was not supported (2/18). Also includes how materials should only be an adjunct to conversations.
