# Supplementary material for: The importance of living well now and relationships: A qualitative study of the barriers and enablers to engaging frail elders with advance care planning
Source: Palliat Med. 2021 May 3;35(6):1137–47. doi: 10.1177/02692163211013260 (PMC8189003; doi:10.1177/02692163211013260)
Supplement: sj-pdf-6-pmj-10.1177_02692163211013260 – Supplemental material for The importance of living well now and relationships: A qualitative study of the barriers and enablers to engaging frail elders with advance care planning [file sj-pdf-6-pmj-10.1177_02692163211013260.pdf]

Supplementary material 6: New practice guidelines supported by the research

KING'S College LONDON | FUNDED BY NIHR National Institute for Health Research | Greenwich & Bexley Community Hospice | StChristopher's More than just a hospice

Strategies for engaging older people living with frailty with advance care planning during COVID-19

Older people living with frailty (older people) are extremely vulnerable to sudden health deteriorations, have a reduced ability to recover, and are at significant risk of contracting COVID-19. This makes engaging older people with ACP a priority. Usual engagement strategies for example, developing trusting relationships and holding conversations over time, are unlikely to be possible in the current situation. So how do we have meaningful, compassionate, yet effective ACP conversations? The six strategies below, adapted for COVID-19, appear to be important in engaging older people with ACP. Suggested phrases should be adapted to the person and context.

**Start early**	This gives the older person the greatest chance to engage physically and cognitively, at their own pace, and to make and revise decisions over time. Starting early during the pandemic is even more important as older people can become ill and deteriorate more quickly than anticipated, with delirium a real possibility.
**Prepare**	Tell the older person you want to discuss their future care preferences in your next conversation. Provide them with any useful, brief ACP resources. Ask them to begin thinking about goals and preferences for their future care and any questions they have for you. If they already have an ACP in place, ask them to start thinking about if the plan is still appropriate in view of COVID-19.
**Relationships**	Relationships are important. Encourage family involvement as desired by the older person. Suggest the older person speaks with their family/those important to them about their wishes before they speak with you. Suggest a family member attends the ACP conversation. Ask the older person if they have a surrogate decision maker or would like help to decide on one. If possible, clinicians should conduct ACP conversations with older people they already have a trusting relationship with. If this is not possible rapport and trust need to be developed swiftly. If you do not know the person, it may be useful to also discuss ACP with their wider care team.
**Living well now**	Older people are often more interested in living well day to day than planning for the future. Ask them *"What is important to you now?"* This can be used as both a preparation question and within ACP conversations. Parallel planning may also be useful. After asking "*What is important to you now?*" or a similar phrase (hoping for the best), ask them about planning for the worst, for example, *"If you were to contract COVID-19, what would you like to happen?"*
**Approach**	Older people want ACP to be treated as normal conversations. Be honest but be gentle with your language, use direct but open questions, for example *"What is your understanding of how COVID-19 is affecting people like you?", "If you were to become seriously unwell due to an illness such as COVID-19, how would you like to be cared for?"*<br><br>Some suggested COVID-19 based scripts are linked overleaf. These are not specific to older people but may be helpful in developing your approach. Following is a script adapted from Vital Talk, a USA based clinician communications platform: (https://www.vitaltalk.org/guides/covid-19-communication-skills/)<br><br>PTO

© King's College London

**Approach Cont.**	○ **Check in:** Ask "*How are you doing with all this?*" Take their emotional temperature<br>○ **Ask about COVID:** *"What have you been thinking about COVID and your situation?"*<br>○ **Lay out issues:** *"Is there anything you'd like us to know if you contracted COVID?"*<br>○ **Motivate them to choose a proxy and talk about what matters:** *"Who could help us make decisions if you weren't able to?" "We're in an extraordinary situation, given that, what matters to you?"* If you think they would be ready to hear it, make a recommendation *"Based on what I've heard, I'd recommend x, y, and z. What do you think?"*<br>○ **Expect emotions:** Acknowledge emotions for example "*This can be hard to think about*".<br>○ **Record the discussion:** Ask for permission to record outcomes in their medical records and any shared electronic medical records systems.<br><br>Approach includes explaining honestly, but gently, why ACP is relevant to the older person. This may include discussing their likely trajectory, the impact of contracting COVID-19, and realistic medical treatment outcomes. Current guidance is that people who are mildly frail or above (Clinical Frailty Score 5 or above https://bit.ly/2UXtpgC), are less likely to benefit from critical care. Discuss whether the older person would wish to be admitted to hospital for any of their other long-term conditions as well as COVID-19 related illness, particularly if having people around them is important as the rules on hospital visiting are unlikely to change for some time.
**Understand**	ACP can be confusing. COVID-19 has highlighted these confusions, particularly around DNACPR leading to treatment withdrawal. This can lead to confused and fearful conversations. Use the phrase "Do not attempt cardiopulmonary resuscitation" rather than "Do not resuscitate/attempt resuscitation". Try to discuss "treatment options" rather than "ceilings of care". You may need to be explicit about other potential treatments options, for example fluids, antibiotics or oxygen. Try not to use jargon or euphemisms, these can lead to confusion, upset and potentially longer or repeat conversations. Check back to ensure their understanding and also summarise the main points to check your understanding.
**Plan**	Use the above strategies, ensure the person has everything they need to make an informed decision. Correct any misconceptions, explain any confusions, and if family were not involved in the conversation, ask how you can support the older person to discuss their ACP decisions with them.

Other COVID-19 scripts:

- **Real Talk** https://www.realtalktraining.co.uk/app/uploads/2020/03/COVID-19-Evidence-based-advice-for-difficult-conversations-2.pdf
- **Respecting Choices:** https://respectingchoices.org/covid-19-resources/
- **Serious Illness Care Programme:** https://covid19.ariadnelabs.org/2020/04/15/serious-illness-care-program-covid-19-response-toolkit/#outpatient-resources

Guidance for patients

- Planning for your future care: A guide (2009) https://bit.ly/3axTvNY
- St Christopher's Advance Care Planning guide (2019) https://bit.ly/2UYN8g0

This document presents independent research funded by the National Institute for Health Research (NIHR). The views expressed are those of the author(s) and not necessarily those of the NHS, the NIHR or the Department of Health and Social Care.
